# A proposal for simplifying the labelling of sunscreens

**DOI:** 10.1111/ics.70103

**Published:** 2026-04-26

**Authors:** Brian Diffey

**Affiliations:** ^1^ Translational and Clinical Research Institute (Dermatology) Newcastle University Newcastle upon Tyne UK

**Keywords:** claim substantiation, computer modeling, evaluation, labelling, safety testing, sunscreen

## Abstract

Determination of sun protection factors (SPFs) using the ‘gold standard’ in vivo method (ISO 24444) is known to be associated with appreciable uncertainty because of experimental variability. Consequently, several in vitro methods have been evaluated, that show promising accuracy, reproducibility and correlation with the in vivo ISO 24444 method. As a way of eliminating the uncertainties associated with both in vivo and in vitro methods, a proposal is made here to dispense with the concept of the SPF and instead to introduce a binary system for labelling the protection afforded by topical sunscreens. Phototesting can be either in vivo or in vitro, with in vivo phototesting requiring only a single area of sunscreen‐protected skin to be irradiated. Alternatively, a validated in vitro method could be employed to determine the threshold protection required for assigning products to the appropriate binary category.

## Graphical abstract

A binary system for classifying the magnitude of protection provided by topical sunscreens is proposed and examples of potential symbols for labelling sunscreens are illustrated here. Phototesting is limited to a single area of sunscreen‐protected skin and avoids assessment of the minimal erythema dose on sunscreen‐protected skin.

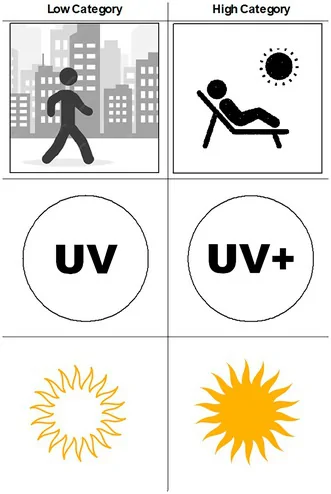

## Résumé

La détermination du facteur de protection solaire (Sun Protection Factors, SPF) à l'ide de la méthode *in vivo* dite « de référence » (ISO 24444) est connue pour être associée à une incertitude notable en raison de la variabilité expérimentale. Par conséquent, plusieurs méthodes *in vitro* ont été évaluées et présentent une précision, une reproductibilité et une corrélation prometteuses avec la méthode *in vivo* ISO 24444. Afin d'éliminer les incertitudes associées aux méthodes *in vivo* et *in vitro*, il est proposé ici d'bandonner le concept de SPF et d'introduire à la place un système binaire pour l'étiquetage de la protection offerte par les écrans solaires topiques. Les essais photobiologiques restent réalisés *in vivo*, mais une seule zone de peau protégée par le produit doit être irradiée. En outre, la méthode évite la nécessité d'évaluer la dose d'UV correspondant à un érythème minimal sur une peau protégée par un écran solaire, un critère d'évaluation souvent incertain et potentiellement sujet à biais. À défaut, une méthode *in vitro* validée pourrait être utilisée pour déterminer le seuil de protection requis afin d'ffecter les produits à la catégorie binaire appropriée.

## INTRODUCTION

Following the testing of 20 popular sunscreens on the Australian market, 16 of them failed to meet their expected sun protection factor (SPF) [1] leading to raised concerns about the reliability of labelled SPFs and action being taken by the Therapeutic Goods Administration [2].

The wide variability in the results of in vivo SPF testing has been known for some while [3, 4] and most recently been demonstrated in an elegant study by Cole et al. [5]. These studies highlight the fact that the inter‐laboratory variability of SPF results is considerable and is likely greater than expected by the public and regulatory bodies. It should be stressed that the huge variation in laboratory testing is under ideal conditions; every volunteer has the same quantity of sunscreen applied to the test areas (2 mg cm^−2^), the sunscreen is spread carefully with a gloved finger, and an international protocol [6] for testing sunscreens is followed.

New in vitro SPF test methods have been investigated to replace the ‘gold standard’ in vivo test and recently two alternative methods were published: ISO 23675:2024 an in vitro method [7] and ISO 23698:2024 [8] a non‐invasive in vivo/in vitro hybrid method. As yet, neither has replaced the ‘gold standard’ in vivo test to become the accepted new standard [9].

In real life nothing like this care is taken when applying sunscreen; variable amounts are applied depending on individual preference and sunscreen is often spread haphazardly and non‐uniformly [10]. What we can conclude from this is that the numerical value of the labelled SPF is a meaningless number in terms of the degree to which users are protected.

## A BINARY CLASSIFICATION OF SUNSCREEN PROTECTION

When it comes to whether sunscreens are permitted to claim broad spectrum, or UVA, protection a binary system has been adopted both in the US and Europe. In the United States, the Food and Drug Administration has adopted a pass/fail test using the in vitro critical wavelength as the sole method for assessing UVA or broad‐spectrum protection [11], whilst in Europe, the European Commission has recommended a minimum ratio of UVA‐PF to SPF of at least one‐third [12] for all sunscreen products marketed in the European Union to claim broad spectrum protection and to be permitted to display an approved symbol to illustrate this.

To circumvent the challenges posed by determination of the SPF, a proposal is made for a binary system for classifying the magnitude of protection provided by sunscreens. The situation that now prevails with a wide choice of SPF is not in the interest of consumers, many of whom do not understand this metric [13] and become anxious and confused about what SPF they should choose.

With more than 4 in 10 adults in the United Kingdom struggling to understand health content written for the public [14], I would argue that consumers do not want to make choices about something they don't understand but fear may nonetheless be important to their health, and that a simple binary protection choice is in their interest. A product that satisfies the criterion (vide infra) for greater protection might be selected by someone who is especially susceptible to sun damage (skin types I and II), who wishes to minimize the UV exposure on their skin with the expectation of reducing the risk of chronic effects, and/or is intending to spend an extended period in a high solar environment.

On the other hand, consumers who are only concerned about casual sun exposure for short periods, who tolerate sun exposure well, who are concerned about the potential for adverse consequences from UV filters either to themselves (e.g. systemic absorption) or the environment (e.g. impact on marine ecosystems), or who simply wish to acquire tanned skin, may opt for a product offering lower protection.

Possible ways in which sunscreen containers could be labelled are illustrated in Table 1, with the appropriate symbol being displayed alongside text such as ‘minimal protection’/‘maximal protection’ or ‘low protection’/‘high protection’.

**TABLE 1:** Examples of potential symbols for labelling sunscreens.

Low category	High category	Comment
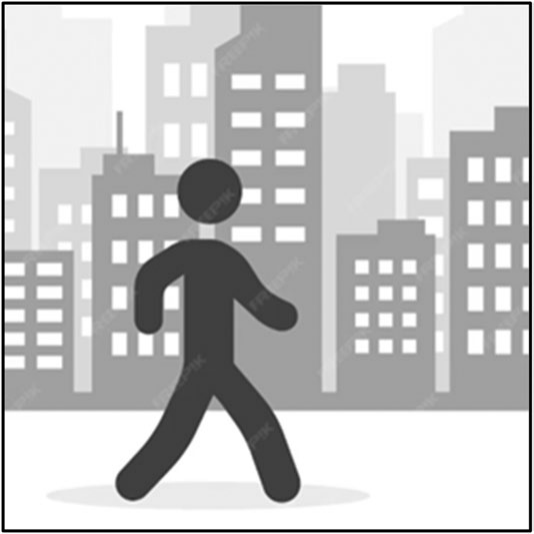	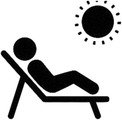	These symbols designate either day‐to‐day casual sun exposure or elective, high dose sun exposure
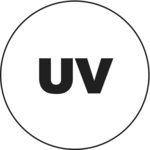	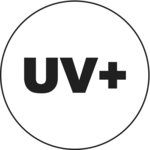	These symbols designate either good UV protection or extra UV protection reflecting the active ingredients and nature of the formulation
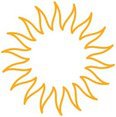	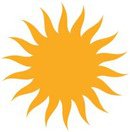	These symbols designate either limited time outdoors and/or low UV index, or extended time outdoors, especially under a high UV index

## THE CRITERION FOR CLASSIFYING SUNSCREEN PROTECTION

The criterion for deciding into which of the two categories a sunscreen should be placed is largely arbitrary. The approach adopted here in choosing the boundary is based on a computational model that allows time‐varying erythema and acclimatization to be determined following repeated exposure to solar UV radiation [15]. The model has previously been applied to examine the effectiveness of sunscreen resulting from spending several days in an environment of high insolation [16].

We consider the personal UV exposure of people with the greatest UV‐sensitive profile (sun‐reactive skin type I) outdoors on each day of a 14‐day vacation under a cloudless sky in mid‐summer from 10:00 to 17:00 at a latitude of 40°N, with a break indoors between 13:00 and 14:00, having lunch, for example. Solar noon occurs at 14:00 with a maximum UV Index of 10. We assume that the exposed skin receives one‐third of ambient UV, reflecting a mix of upright and reclining postures [17].

A sunscreen that binds well to the skin is applied at a thickness of 1 mg cm^−2^ reflecting typical usage [10] at 2‐hourly intervals starting at 10:00 and ending at 16:00. Because of the mismatch between the spectral emission of sunlight and that of solar simulators used in the determination of SPF, delivered SPFs in sunlight are lower than those obtained in the laboratory both from a theoretical [18] and experimental [19] perspective, and this was considered in the calculations, as has been adopted previously [16].

The aim is to ensure that under these conditions of sun exposure and sunscreen application nothing more than a mild erythema, which we define as a slight colouration of the skin with no pain or discomfort, is expected to result at the end of the 14‐day period. Calculations were repeated for broad‐spectrum products labelled SPF15, SPF30 and SPF50.

Figure 1 illustrates the time course of the calculated erythemal response over this period. We see that daily application of an SPF15 product every 2 h whilst outdoors is predicted to result in moderate erythema after 1 week or so but that both SPF30 and SPF50 result in only mild erythema by the end of the 2‐week period with only a small difference in predicted response between these two SPFs. For people with sun‐reactive skin types II or higher, we would predict an even smaller erythemal response.

**FIGURE 1:**
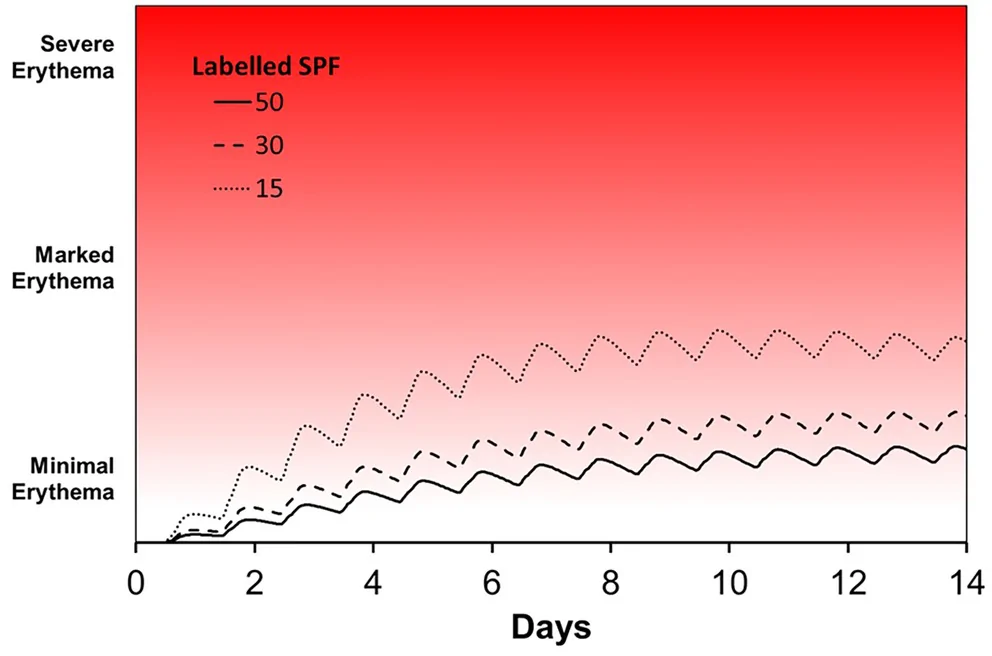
The predicted erythemal response over a 14‐day period in a subject of skin type I in which sunscreen of labelled SPF15, SPF30 or SPF50 has been applied at 2‐hourly intervals from 10:00 to 16:00 at an average thickness of 1 mg cm^−2^. Sun exposure occurs on each day of a 14‐day vacation under a cloudless sky in mid‐summer from 10:00 to 17:00 at a latitude of 40° N, with a break indoors between 13:00 and 14:00. Erythema is indicated qualitatively with no apparent redness at doses less than 1 MED, increasing through pale pink to deep red as erythema progresses from minimal through mild, moderate and marked to severe.

We consider now a more extreme example of a sun‐seeking holiday in the tropics where solar noon occurs at 13:00 with a maximum UV Index of 13, sun exposure occurs continuously from 10:00 to 17:00 and calculations are repeated for SPF30 and SPF50 products. The results illustrated in Figure 2 indicate that both products could well lead to moderate erythema, although applying the SPF30 more generously at an average thickness of 1.4 mg cm^−2^, as might be the case for a product that is pleasant and easy to apply, provides slightly better protection than an SPF50 applied at 1 mg cm^−2^. Also, applying the SPF50 more thinly at an average thickness of 0.7 mg cm^−2^, as might be the case for a product that is not especially pleasurable to use, provides less protection than an SPF30 applied at 1 mg cm^−2^.

**FIGURE 2:**
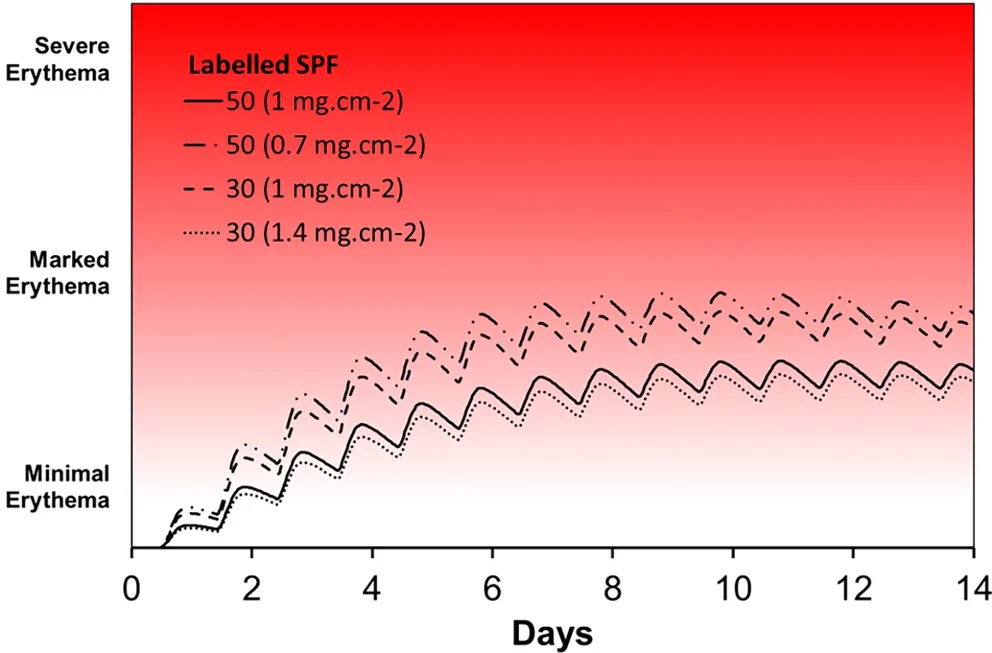
The predicted erythemal response over a 14‐day period in a subject of skin type I in which sunscreen of labelled SPF30 or SPF50 has been applied at 2‐hourly intervals from 10:00 to 16:00. Sun exposure occurs in the tropics without a break on each day of a 14‐day vacation under a cloudless sky in mid‐summer from 10:00 to 17:00.

## EVALUATING SUNSCREEN PRODUCTS

Because we only need to know whether a product exceeds SPF30 when tested in the laboratory, there is no need to determine the precise SPF and either an in vivo or in vitro technique could be employed.

If in vivo phototesting is chosen, an assessment would be made of the predicted SPF based on the concentrations of the active UV filters, using an online sunscreen simulator* and phototesting would only proceed if the predicted SPF exceeded 15. The reason for calculating the SPF rather than estimating it by an in vitro technique, which requires specialized equipment, is simply to avoid excessive erythema developing in volunteers when they are exposed to a single dose of UV.

As is current practise with ISO 24444 [6], each member of the volunteer panel would be exposed to an increasing series of UV exposures on unprotected skin and 24 h later would return to have their minimal erythema dose (MED) determined. Sunscreen at a thickness of 2 mg cm^−2^ would be spread uniformly over a single test site, which is then exposed to a UV dose equal to 30 times their MED.

The skin would be inspected a further 24 h later and a note made of any erythema seen at the test site, ideally by someone not involved in the phototesting. The degree of erythema is of no consequence—redness is either present or absent. This approach avoids the requirement to assess the UV dose corresponding to a minimal erythema on sunscreen‐protected skin, an endpoint that is often uncertain and can lead to ‘expectation bias’.

For a product to be placed in the higher protection category, we offer the pragmatic proposition that erythema would have to be absent in 80% or more of the volunteers; otherwise, the product would be labelled corresponding to the lower protection category.

In determining SPFs in a volunteer panel according to ISO 2444, a product with an expected SPF of 30 will result in a spread of individual SPFs with a standard deviation of around 6 or so. If we follow ISO 2444 [6] and assume individual SPFs are normally distributed, the mean SPF will need to be 35 or higher for 80% or more of volunteers to have individual SPFs greater than 30.

As an alternative to in vivo testing, the validated ISO 23675:2024 in vitro technique [7] could be utilized and products assigned to the appropriate protection category according to the SPF30‐equivalent result.

## DISCUSSION

A novel method is proposed for labelling sunscreens. It is a departure from the concept of the SPF, which was popularized by the Austrian scientist Franz Greiter in the 1970s and subsequently adopted by many regulatory authorities and the cosmetic and pharmaceutical industries, and remains the ‘gold standard’ for assessing the magnitude of protection afforded by topical sunscreens.

However, it is argued that the binary system proposed here for choosing a sunscreen eliminates the need for multiple choices and is in consumers' interest, especially as the numerical protection factor, that is, SPF, is a meaningless guide to the level of protection delivered. This is especially true for those who interpret the SPF as how much longer skin covered with sunscreen takes to burn compared with unprotected skin.

We have defined an arbitrary set of exposure conditions involving a fixed latitude of 40° N (which defines the peak UV index), a cloudless sky over 14 consecutive days, time outdoors, body posture leading to the assumption of one‐third fraction of ambient UV received by the skin, skin type, sunscreen application thickness and reapplication frequency to arrive at an SPF that is predicted to result in nothing more than mild erythema after 14 days of sun exposure. There are an infinite number of other combinations that could have been chosen but we argue that the conditions selected reflect sun‐seeking behaviour in someone with sensitive skin. There are, of course, more extreme exposure scenarios, as illustrated in Figure 2.

From the results of modeling, we arrive at the conclusion that products whose laboratory‐determined SPF exceeds 30 can be placed in the higher protection category, with products not reaching this threshold assigned to the lower category. This threshold reflects current advice from several agencies including The World Health Organization, the American Cancer Society, the UK National Health Service and the British Association of Dermatologists, who all recommend using a broad‐spectrum sunscreen with a minimum SPF of 30 for those wishing to protect against skin cancer and UV damage.

Whilst SPF50 sunscreens deliver greater protection than SPF30 when applied at the same thickness, it is clear from Figure 2 that a more generous application of the lower SPF product more than compensates for the lower concentration of active ingredients. Good aesthetic properties (appearance and skin feel) are an important aspect of sunscreen products, as they encourage greater consumer compliance. Consequently, those products with good sensory properties are associated with a subjective assessment of a pleasurable product, which results in higher application thicknesses and hence greater delivered photoprotection than products with negative attributes such as greasiness or leaving a white film on the skin, and confirm that the benefit associated with sunscreen use is strongly linked to cosmetic attributes and compliance as much as, or even more than, the SPF [20, 21].

Nevertheless, in situations of prolonged exposure under high UV levels, Figure 2 illustrates that sunscreen alone is likely to be insufficient to prevent sunburn in white skin and that shade and/or clothing should be considered in addition to sunscreen [16].

Realistically, despite the advantages claimed for this new proposal, the inertia associated with historical practise means that it is unlikely to be realized. Nevertheless, it is hoped that, if nothing else, the proposal encourages us to take a step back and re‐evaluate whether the current methods, either in vivo or in vitro, of trying to determine varying magnitudes of protection against a backdrop of significant uncertainty should remain our goal.

## FUNDING INFORMATION

The author has nothing to report.

## CONFLICT OF INTEREST STATEMENT

The author declares no conflicts of interest.

## Data Availability

Data sharing not applicable to this article as no datasets were generated or analysed during the current study.

## Footnotes

* For example, https://care360.basf.com/global/en/industries/personal‐care/digital‐services/d‐lite‐‐‐‐sunscreen‐simulator.
